# Ethical Issues in Fetal Management: A Cardiac Perspective

**DOI:** 10.1155/2010/857460

**Published:** 2010-03-24

**Authors:** Atul Malhotra, Samuel Menahem, Lynn Gillam

**Affiliations:** ^1^Department of Neonatology, Monash Medical Centre, 246, Clayton Road, Clayton, Melbourne, Victoria 3168, Australia; ^2^Fetal Cardiac Unit, Monash Medical Centre, 246, Clayton Road, Clayton, Melbourne, Victoria 3168, Australia; ^3^Departments of Pediatrics & Psychological Medicine, Monash University, Melbourne, Victoria 3168, Australia; ^4^Murdoch Children's Research Institute, Melbourne, Victoria 3052, Australia; ^5^Children's Bioethics Centre, Royal Children's Hospital, Melbourne, Victoria 3052, Australia

## Abstract

The ethical issues behind the management of a fetus with a serious abnormality and the decisions made in relation to the outcome of the pregnancy are complex. This reflective paper deals with the ethical principles of managing a pregnancy with a congenital anomaly, with particular emphasis on the fetus with a serious cardiac abnormality. One major ethical concern is whether the fetus is or is not independent being to whom obligations of beneficence are owed. We review the debate on this matter, and suggest that it is ethically more appropriate for physicians who are involved in management of fetal abnormality not to adopt and insist on their own position on this matter. Rather, the appropriate course is to respect the pregnant woman's own view of her fetus and how it should be regarded. This is an application of the principle of respect for autonomy. Within this framework, we discuss the difficulties in counselling a pregnant woman or expectant couple in this situation, and recommend three key steps in ethically sound counselling.

## 1. Introduction

Prenatal diagnostic ultrasound is widely performed especially in the western world. Parents attending a scan expect to be told that their baby is normal. They want to be reassured regarding the size and well-being of the infant, and may wish to know its sex [1]. When a possible anomaly is identified, it comes as a shock to the parents, who were not expecting such an outcome. If the anomaly is subsequently confirmed, there may be an assumption that as the parents sought screening in the first place; they intended to proceed to an abortion [2]. However, this is not always the case, given their expectations at the outset. Counseling the parents in such situations is complex and requires much sensitivity. This paper reviews the ethical issues involved and makes recommendations for practice.

## 2. Basic Ethical Principles

Chervenak et al. have very eloquently described the basic ethical principles in the management of pregnancies complicated by fetal anomalies [3]. The first ethical concept is that of beneficence. Health related interests of the patient obligate the physician to seek clinical benefits over clinical harms for the patient. The second basic ethical principle is respect for the patient's autonomy. This principle means that the patient's perspective on health-related and other interests is paramount. The physician needs to respect the patient's own set of values, beliefs and decision-making capacity. The physician's role is to provide adequate information and a recommended management plan, or range of possible plans, for the condition in question. It is vital that the information is provided in a manner that allows the patient to understand it, so as to be able to reach an informed and voluntary decision. We will discuss the physician's ethical obligations in relation to informed consent in more detail below. However, it is important to note that the expectant parents' great state of distress, grief or shock may make it very difficult for them to take in, understand and assimilate what is provided [4], even with very careful presentation of information 

The crucial ethical question in pregnancies complicated by fetal anomalies, according to Chervenak et al. [3], is whether the fetus counts independently as a patient to whom the obligations of beneficence are also owed (in addition to the pregnant woman). If the fetus is also a patient, then the ethical situation becomes much more complex. What is best for the pregnant woman may differ from what is best for the fetus (for example, where there are physical risks to the woman in continuing the pregnancy). In addition, the woman may make decisions which are contrary to the best interests of the fetus (for example to terminate a viable pregnancy). Difficult choices may have to be made and are based on where ethical priorities lie.

There are two possible approaches to dealing with the question of whether the fetus should be regarded as a patient. The first approach is that the physician comes to his or her own moral decision about whether the fetus should be regarded as a patient. This approach would require the physician to have a clear and sound moral or philosophical basis on which to make this decision. The second approach is that the physician adopts no view and leaves it up to the pregnant woman (and her partner, if involved) to decide how they wish to regard their fetus. This approach need not involve any moral or philosophical reasoning by the physician about the fetus; simply giving primacy to the pregnant woman's autonomy in relation to decisions relating to her fetus. 

Chervenak et al. [3] suggest a variation of the first approach. They concede, as many others do [1, 5], that the fetus cannot meaningfully possess values and beliefs, and is therefore not a person to whom obligations can be owed. However, they maintain that the obligations of beneficence to the fetus arise from the fact that obligations are owed to the infant which that fetus will become after birth. This makes the fetus a patient, regardless of whether it is a person. More specifically, Chervenak et al. argue that the fetus becomes a patient only after viability; the pre-viable fetus does not have the status of a patient, and should only be treated as a patient if the pregnant woman wants to regard it as such. Once a fetus is viable (a state related to the biological stage of development aided by the availability of medical technology) it is possible for the fetus to survive independently outside the womb. Hence, according to Chervenak et al., there are beneficence obligations to the viable fetus, whenever it is presented to the physician and there exist medical interventions (whether diagnostic or therapeutic) that could. produce a greater balance of clinical good over clinical harm for it in the future that is, when it becomes an infant, a child or an adult. There is extensive data to support the possibility of clinical benefit in cases of cardiac anomalies [6–8].

If this argument is accepted, it means that the physician may end up having an obligation to seek to change or override pregnant woman's wishes, for the sake of the fetus. If the physician believes the fetus is a patient, owed the same obligations as any other patient, then his or her obligation is directed to the best interests of the fetus. If the pregnant woman's decisions are contrary to the best interests of the child that the fetus will become, then the physician has an obligation to protect those interests, just as for any child put at risk by parental decisions about medical treatment. The logic of Chervenak and his co-authors' position implies that if attempts at persuasion do not work, the physician may have to seek legal avenues to override the woman's decision, a course of action that could lead to court-enforced fetal surgery [9], immediate delivery of the fetus and, in theory, court-ordered continuation of pregnancy (although it should be noted that in situations where the local laws permit abortion, they do not generally allow for a third party to prevent a woman having an abortion). 

However, we caution against this approach, where the physician adopts an independent moral stance on the fetus, and seeks to act accordingly. Whilst the arguments of Chervenak et al. [3] are well reasoned, there are also well-reasoned arguments to the opposite effect, namely that the fetus should not at any stage of gestation be regarded as a patient to whom the physician has direct obligations, unless the pregnant woman chooses to do so. The obligation to the fetus, as Chervenak et al. acknowledge, is based on the well-being of the child it will become. However, whether or not the fetus becomes a child depends on the woman continuing with her pregnancy. It could be argued that if she decides to terminate her pregnancy, at any stage and for whatever reason, there is no longer any obligation to the fetus, since there will not be any child. This conclusion is contrary to the view of Chervenak et al., yet draws on the same reasoning they do. 

The well-known difference in views about the status of the fetus and the morality of abortion, across different cultures and religions also introduces a note of caution. A physician working in the multi-cultural setting of today's increasingly globalised world is likely to encounter patients with quite varied views. In addition, laws relating to abortion vary considerably between jurisdictions. We suggest, then, that the second approach suggested above is preferable, namely for the physician to leave it to the pregnant woman (and partner) to decide if the fetus is to be regarded as a patient or not (providing that local laws permit abortion). 

Adopting that view does not mean that the physician should not have a personal position on the status of the fetus, only that he or she should not attempt to impose it on his or her patients. If the wishes of the pregnant woman in regards to termination of her pregnancy or intra-uterine therapy for her fetus are significantly at odds with the physician's moral views, the physician should exercise the right to conscientious objection, and hand over the care of the patient to another doctor [10]. This obligation to refer is a standardly accepted caveat on the right to conscientious objection [11]. The Australian Medical Association Code of Ethics [12], for example, states the following: 

When a personal moral judgement or religious belief alone prevents you from recommending some form of therapy, inform your patient so that they may seek care elsewhere, and recognise your right to refuse to carry out services which you consider to be professionally unethical, against your moral convictions, imposed on you for either administrative reasons or for financial gain or which you consider are not in the best interest of the patient. 

This position is an attempt to negotiate between competing moral values: the woman's autonomy and the physician's integrity. The physician is not forced to do something he or she believes morally wrong, but the woman is also able to exercise her own choice. 

## 3. Ethical Responsibility of Cardiologists When a Serious Fetal Cardiac Anomaly Is Found

Physicians working in obstetrics and gynaecology are presumably aware of the need to work through these issues of the status of the fetus, and to develop their position on abortion, as these issues form a major aspect of their practice. Paediatric cardiologists, on the other hand, have had little cause to consider such issues when working in their discipline, and may never need to do so. However, since it is now possible to detect fetal cardiac anomalies prenatally, cardiologists are coming face to face with these issues. There is usually (in most jurisdictions) an option to terminate an affected pregnancy and increasingly intrauterine interventions may be possible. Cardiologists must consider how they will counsel women in these situations, how directive they will be about which option should be chosen, and what they will do if the woman's choice is not the one that optimizes life and health for the fetus.

Here, we set out the key steps in the counseling process from an ethical perspective, and make recommendations about ethically appropriate practice. These steps may take place over more than one consultation, and may need to be re-visited on each occasion, due to the emotional nature of the situation and the complexity of the information to be conveyed.

### 3.1. Step  1. Give Accurate Information about the Diagnosis and Prognosis of the Cardiac Abnormality

The first stage is providing accurate information about the diagnosis and prognosis in a manner and at a timing that the expectant parents are able to understand. Fetal cardiac anomalies like any congenital anomaly necessitate that physicians provide the parent adequate information. For any counselling to be credible the diagnosis must be accurate. This is even more relevant in the case of antenatally diagnosed cardiac anomalies. The general screening detection rates for congenital heart disease (CHD) vary between 14%–45% [13]. A standard 4 chamber view can detect 40%–50% of major CHD [14], while a 4 chamber view and outflow tract detects 70%–80% of major CHD [15]. In dedicated fetal cardiac centres the diagnostic accuracy is close to 100% [4, 16]. Fetal cardiac malformations are compounded by the fact that other malformations may be present, as is the possibility of chromosomal abnormalities. The most accurate information possible should be given to the expectant parents, along with a clear explanation of what is still uncertain, unclear or subject to change as the pregnancy progresses. The physician should keep in mind the possibility of evolving lesions [10] (e.g., a developing left and right hypoplastic heart syndrome) and inform the parents accordingly. For such condition, there is inadequate or incomplete data as far as their outcome and natural history, and this also must be conveyed to the parents.

Shinebourne argues that most CHD are treatable with a resultant reasonable quality of life [5]. Even in serious cardiac conditions, one is not always able to clearly define the possible outcomes. Few cardiac conditions are not amenable to at least palliative surgery, if not complete repair. In most cases the neurological development is normal or close to normal [17]. When dealing with fetal anomalies detected on ultrasound, the questions and concerns raised by parents relate to the quality of life issues starting from infancy right up to adulthood [18]. Generally the details of the abnormality, while important, are not the paramount issue for the parents [19]. Complications of the abnormality and the results of surgery or any intervention also figure in the considerations. There is the need to describe possible poor outcomes, especially if they are severe even though unlikely to happen. This information allows the parents to decide how to proceed with knowledge of the worst case scenario [10]. 

The physician needs to ensure the expectant parents understand the information about the nature of abnormality, the implications for the life of the future child, the possibility of intervention, and the risk for each intervention prenatally or postnatally. Parents also need to know the figures for local practice, for short, medium and long term outcomes, especially with respect to quality of life issues. The physician must be ready to discuss all of these issues with parents, providing the best available information, but also indicating the limits and uncertainties in this information and at a time when the parents are able to take in the information.

### 3.2. Step  2. Identify Options

The next stage is to identify and present the options available. In brief, there are three main options: to continue with the pregnancy, to terminate the pregnancy (if legally permitted), or to consider prenatal intervention (if it is possible for the condition and available). If the decision is to continue with the pregnancy, there will perhaps be further decisions to make as to where the infant is to be delivered, the need for in utero transfer, and the mode of delivery [19]. There will also need to be an anticipatory management plan for the infant after birth. Parents will generally accept what is recommended to them on these matters, but still require them to be explicitly stated. If the decision is to terminate, there may be a need to shift hospitals (from example from a Catholic hospital), or change the obstetrician if termination is not personally acceptable to him or her. The parents should be made aware of these implications, not in an attempt to change their mind, but to inform and prepare them for the process.

Local laws and practices play an important role in the decision making. For example in some places it may be legal to terminate a pregnancy for maternal psychosocial reasons [17]; in other places, fetal indications may be specified in the law. There may or may not be restrictions on termination related to the stage of gestation. Broadly speaking, obstetricians are able to carry out a termination before 12 weeks [20], but the risk of legal complications increases after 12 weeks and especially after 20 weeks (which is about the stage at which antenatal diagnoses of cardiac anomalies are more commonly made.) In our state of Victoria, in Australia, the law has recently changed to allow termination for any reason up until 24 weeks, and after that there is the need for two doctors to agree that it is reasonable. Physicians must develop an accurate understanding of their local laws and seek legal clarification if necessary. 

During the counselling, assuming a “neutral” tone on the part of the clinician—not overly pessimistic or optimistic—is vital [2] but may be extremely difficult to achieve. The ultimate aim is to allow the expectant parents to form their own assessment of the impact the condition would have on their future child. As Shinebourne [5] notes: “It is the mother's perception of the fetal cardiac anomaly and not the cardiologist's that should determine outcome (continuation or termination)”. It is open to question, though, how achievable this is in a setting of acute emotional distress where the mother is in a state of shock and grieving the loss of a sought-after normal infant [4]. The ethical obligation is to do one's best to achieve this aim.

### 3.3. Step  3. Discuss Options

The next stage is discussing the options with parents which is the most ethically contentious stage. There are different views even about which matters are ethically appropriate to raise and discuss, let alone about the degree to which it is appropriate to recommend or favour a particular option, rather than being as neutral as possible. Most professionals advocate non-directive [4, 5, 21] counselling if possible. It is important to realize that the impact of counseling is affected by the physician's approach, speech, tone, and so forth [5]. In many counseling sessions, selective information is provided, whether deliberately and inadvertently, though some feel obligated to provide all the information available. There is also the question as to who is the best person to do the counseling. Cardiologists, genetic counsellors or obstetricians have counseled independently or together [4].

Making a decision may not be easy for the parents. They have to come to terms with the abnormality and grieve the loss of a normal infant, as well as grapple with the questions of what they think about abortion, disability, their personal capacity to care for such an infant/child and their ideas about parenthood and family life. They may wish to talk through the options. They may want their cardiologist's opinion about what they should do. Simply giving such an opinion may not be the best option as the personal circumstances of the clinician differ from that of the parents. It is preferable to discuss how one might decide, what factors one would take into account, in order to model to the expectant parents a way of thinking about the issue, rather than simply give them an answer.

The reasons for considering termination may be very variable, complicated and as Shaffer et al. [22] acknowledge, may not necessarily be “rational” in the strictest sense. This can make the discussion of options difficult, especially for those not specifically trained for these situations. The reasons for which women decide to terminate affected pregnancies are not well documented or understood, though the few studies done in the area indicate it is the nature of condition rather than the stage of gestation that carries most weight [23]. A common understanding of physicians is that most terminations for fetal cardiac anomaly are done to minimize distress and grief to the parents of having a child with reduced physical activity who may die young. This allows mother time for other important aspects of her life, care of the other children and to prevent hardship to others [5]. There may be also be other social reasons, or a medical condition of the pregnant woman.

Note that in these discussions, the pregnant woman (and partner) is not necessarily thinking of the the fetus as a baby, or being with independent rights. Ethically speaking, a pregnant woman's decision need not be based on what is best for the future child; she may legitimately considers her own and others' interests [5]. For example, there is the perception that an anomaly may have a traumatic or deleterious effect on the parents and the other children, as well as the future child [24]. Here, it is important to note the differences between expectant parents' decisions regarding termination of pregnancy and actual parents' decisions regarding the treatment of the newborn infant. The actual parents' role is to decide on behalf of the infant, on the basis of what is in best interests of the infant [11]. Once delivered, the priority is on maintaining the life and health of the infant, aiming for the best outcomes. If the parent's wishes are significantly contrary to the infant's well-being they can be legally overridden. In contrast, decisions regarding the termination of the pregnancy are not ethically required to be about the best interests of the future child. If the local laws permit termination of the pregnancy, prospective parents may decide for their own reasons. This *may* include what they perceive would be in best interests of the future child, but will not necessarily do so. 

Specialist prenatal genetic counsellors may be particularly helpful for the expectant parents in talking through these sorts of issues, though such counselors may not have the detailed cardiac knowledge to answer the relevant questions. What parents need most from the paediatric cardiologist is the best available understanding of what their child's life would be like, what sort of interventions would be needed and the risks of these to the child in the local setting. 

## 4. Intrauterine Intervention: Ethical Issues

If intrauterine interventions are available the further important issue is the pregnant woman's autonomy versus the potential beneficence to the fetus and future child, where the pregnancy is going to continue and the fetus can reasonably be regarded as a patient. In this situation, the pregnant woman is in the ethical role of parent making decisions for the health of her future child—but she is also making decisions about herself and her own health. There is a conflict between American College of Obstetricians and Gynecologists and the American Academy of Pediatrics [25] regarding the issue of fetal interventions. The latter accords less weight to maternal decision making and is more tolerant of overriding maternal refusal of intervention which may be suggested for fetal benefit. An intervention to the fetus may pose risks to pregnant woman which she may not be willing to take. One example is prescribing medication to the mother for treating fetal arrhythmias. Another example is surgical intervention for critical aortic or pulmonary stenosis [26–28] which has been employed to improve fetal outcomes, though it may lead to premature labour and may require a surgical incision in pregnant woman to facilitate needling of the ventricles. 

One important question to answer is whether the potential benefit to the fetus warrants the risks to the pregnant woman. Another important question is about the risks to the fetus: are they sufficiently outweighed by potential gains that it is reasonable to expect overall benefit to the fetus? One may argue that it is reasonable to offer and recommend fetal therapy if there is a probability of saving the life of the fetus or to prevent serious or irreversible disease to fetus or child and yet carries a low mortality/morbidity risk to the fetus/child and low morbidity to mother [27]. But when the results of such an intervention are questionable and not without risks to the mother, it is not so straightforward. We suggest that in these situations, respect for the woman's autonomy and her assessment of her own and her future child's health-related interests be given priority.

 Arguably, such innovations as in utero fetal surgery need to be conducted and evaluated as research [29], preferably conducted in centres of excellence. In such cases, the women need be considered as research participants. Genuine voluntariness and informed consent is a standard prerequisite for any such research. 

## 5. Multiple Pregnancies with One Fetus with a Serious Cardiac Anomaly: Ethical Issues

We have discussed this issue in detail in a separate paper [30]. To summarise, dealing with the management of a twin pregnancy where one fetus has a serious anomaly is one of the most challenging and confronting issues a clinician faces. The management depends on the wishes, values and preferences of the mother/parents once provided with detailed and accurate information about the condition of the twins. The risk to the unaffected fetus depends on chorionicity and becomes much more problematic in monochorionic pregnancies. Selective termination of an affected twin with a serious congenital heart disease is possible in some circumstances. Clinicians need to provide detailed probability of the risks of selective termination to pregnant woman and the risks to the normal fetus, including the onset of premature labour, cerebral hypoxia and death to the unaffected twin. It is also important for expectant parents to understand that fetal death of the normal twin, especially in monochorionic twin pregnancies, may occur even if the twin pregnancy is continued, in circumstances where the affected twin becomes unwell or dies.

## 6. Conclusions

The basic ethical principles of respect for autonomy and beneficence play the major role in grounding physicians' ethical responsibilities in pregnancies where there may be a fetal cardiac anomaly. The physician's main ethical obligation is to provide adequate and correct information, in a way that takes account of the extremely distressing circumstances, so as to allow for informed decision making. The ethical principle of autonomy creates a responsibility for the physician to help the pregnant woman make informed decisions based on her values and aspirations. A decision to terminate is partly a medical matter, relating to the procedure and its risks, and partly a personal moral decision bound by legal, cultural and religious constraints. Clinicians can advise regarding the former, but it is not within their realm to advise regarding the latter. The decision whether to continue or terminate in fetal cardiac anomalies is complicated by the fact that in the present era there are very few cardiac conditions which are not amenable to repair and with a reasonable future quality of life. Parents have to deal with shades of grey. There is the additional issue of increasing fetal interventions which may be helpful for the fetus/infant/child but may put the mother at risk and who in turn may opt out of any such intervention. A twin pregnancy with one affected twin further compounds the ethical considerations further. In all situations, the overriding imperative is to provide accurate information about the diagnosis and outcome, identify and discuss possible options and their potential consequences for the fetus and the pregnant woman.
